# Heats of combustion representative of the carbohydrate mass contained in fruits, vegetables, or cereals

**DOI:** 10.1002/fsn3.1175

**Published:** 2019-08-14

**Authors:** Ana‐Guadalupe Martínez‐Navarro, Eulogio Orozco‐Guareño, María‐Judith Sánchez‐Peña, Edgar‐José López‐Naranjo, Priscilla Muñiz‐Mendoza, Luis‐Javier González‐Ortiz

**Affiliations:** ^1^ Chemistry Department, University Center of Exact Science and Engineering University of Guadalajara Guadalajara Jalisco Mexico; ^2^ Projects Engineering Department, University Center of Exact Science and Engineering University of Guadalajara Guadalajara Jalisco Mexico

**Keywords:** carbohydrates, cereals, fruits, Heats of combustion, vegetables, vegetal source foods

## Abstract

The obtainment of suitable values for metabolizable energy requires the previous knowledge of accurate and precise values of the heat of combustion of the different macronutrients. Thus, in this work, the heats of combustion of six carbohydrates (glucose, fructose, sucrose, maltose, starch, and cellulose) were experimentally measured, and such values were statistically compared with equivalent bibliographic values collected in a parallel work (Heats of combustion of the main carbohydrates in vegetable foods: a bibliographic approach, 2019), proposing, for each carbohydrate, an “overall interval” and an actualized representative value, which were estimated considering jointly the bibliographic and experimental information. Besides, a numerical methodology that used such parameters and the relative content of the different carbohydrates in selected foods was proposed, to estimate the global heat of combustion producible by the carbohydrate mass contained in such foods. The results estimated for 68 foods were globalized to propose the following generalized heats of combustion: (a) for fruits: 3.88 kcal/g, (b) for vegetables: 3.98 kcal/g and, and (c) for cereals: 4.13 kcal/g. These results demonstrated that the use of the Atwater's value (4.2 kcal/g of carbohydrate of vegetable source) involves a clear overestimation of the heat of combustion of the carbohydrate mass contained in vegetable source foods.

## 1 INTRODUCTION

Negative consequences in public health caused by the overweight and obesity epidemics occurring in several countries are evident. Among other actions, some nutritional strategies could be used to prevent or avoid them. However, to implement them properly, it is imperative to know, as precise as possible, the energy that the human body is truly capable of obtaining from the different macronutrients contained in its foods and use for its organic functions, that is, the metabolizable energy (${\text{ME}}_{n}$, being n the respective macronutrient). Since the currently used metabolizable energy values (4 kcal/g for carbohydrates *[c]*‐ and proteins *[p]*‐ and 9 kcal/g for lipids *[l]*) were published more than a century ago (Atwater, 1910), it could a priori be recommendable to update such data. Thus, in the first paper of this series (Sánchez‐Peña et al., 2017), an exhaustive critical review of the experiments carried out by Atwater to obtain such values was presented, demonstrating that there is a series of weakness in the experimental design that could compromise the validity of such values.

Regarding carbohydrates, Atwater (1900) proposed that the metabolizable energy of carbohydrates (${\text{ME}}_{c}$) can be estimated as follows: ${\text{ME}}_{c}=\left({\text{HC}}_{c}\right)A_{c}$ *,* being ${\text{HC}}_{c}$, the heat of combustion produced by each gram of carbohydrate and, $A_{c}$, the availability coefficient of such macronutrient. Focusing on the $A_{c}$ values characterizing the vegetal source foods, Atwater and Bryant (1900) proposed the following values for such coefficient: for cereals: 0.98, for legumes: 0.97, for vegetables: 0.95, and for fruits: 0.90; although the deficiencies and information absences related to the obtainment procedure of such factors have been recently summarized (Sánchez‐Peña et al., 2017), implicitly, such factors are still used. Therefore, even recognizing certain inaccuracy level on the $A_{c}$ factors reported by Atwater and Bryant (1900), it could be expectable that such factors are very close to 1.0; thus, the accuracy and precision level of the ${\text{ME}}_{c}$ value characterizing a given sample must be strongly dependent on the accuracy and precision level of its correspondent ${\text{HC}}_{c}$ value. Therefore, as a first step in the estimation of a suitable ${\text{ME}}_{c}$ value, the establishment of a suitable ${\text{HC}}_{c}$ value is required.

With respect to the ${\text{HC}}_{c}$ value, Atwater and Bryant (1900) proposed that, for vegetal source carbohydrates, ${\text{HC}}_{c}$ = 4.2 kcal/g. Nevertheless, since such value was proposed considering an arbitrary “averaging procedure” that probably used inaccurate data (Sánchez‐Peña et al., 2017), it could be considered necessary to reevaluate such parameter.

With such background, in a parallel work (Sánchez‐Peña et al., 2019), an exhaustive bibliographic analysis related to the heats of combustion of glucose, fructose, sucrose, maltose, starch, and cellulose was performed. In this analysis, the experimental errors possibly involved in the original sources were taken into account, and following rigorous statistical arguments, the outlier data were eliminated. In that work (Sánchez‐Peña et al., 2019), the following information was reported for each carbohydrate: (a) a representative value for the heat of combustion of the respective carbohydrate (which could be considered as the best approximation to the real heat of combustion value), and (b) an interval named “bibliographic interval,” which very probably includes the real heat of combustion value. The representative values (expressed in kcal/g) reported by Sánchez‐Peña et al. (2019) were the following: (a) glucose: 3.73, (b) fructose: 3.74, (c) sucrose: 3.95, (d) maltose: 3.98 (monohydrated maltose: 3.75), (e) starch: 4.16, and (f) cellulose: 4.16. Complementarily, Sánchez‐Peña et al. (2019) reported the following “bibliographic intervals”: (a) glucose: 3.69–3.78, (b) fructose: 3.71–3.77, (c) sucrose: 3.91–3.99, (d) maltose: 3.91–4.05 (monohydrated maltose: 3.69–3.82), (e) starch: 4.07–4.25, and (f) cellulose: 4.05–4.26.

Thus, although the previous values and intervals were obtained following a rigorous statistical procedure (Sánchez‐Peña et al., 2019), the possibility of experimental deficiencies in some of the original bibliographic sources makes them potentially questionable. Therefore, the estimation of the agreement level between such parameters and a set of experimental values currently measured could be considered as necessary.

Thus, to reinforce the validity of the previously mentioned bibliographic parameters (first intermediate aim of this work), an experimental procedure to measure the heat of combustion of the carbohydrates of interest was here implemented, where the experimental inaccuracy of the previous works were carefully minimized (see details below); for each carbohydrate, an “overall interval” and an actualized representative value were statistically estimated, considering jointly the bibliographic information collected by Sánchez‐Peña (2019) and the experimental data obtained in this work.

Then, a mathematical procedure was here proposed, which allow to estimate a global representative value by mass unit (global value) and a global interval for the heat of combustion by mass unit (global interval) that could be properly used to characterize to the carbohydrate mass contained in a given food; second intermediate goal of this work. In such procedure, the statistically estimated parameters mentioned in the previous paragraph, as well as the contents of the different carbohydrates contained in the considered vegetal products (obtained from the USDA Food Composition Database (National Nutrient Database for Standard Reference, 2016)) were used.

Finally, the global objective of this work was to propose a final representative value and a global interval that could be used to adequately characterize the heat of combustion per unit mass, corresponding to the carbohydrate mass contained in each food group. For this, the global intervals calculated for each one of the considered foods were grouped, following a procedure proposed here.

## 2 METHODOLOGY

### 2.1 Experimental measurements

#### 2.1.1 Materials

As raw materials, the following carbohydrate samples were used: D‐(+)‐glucose (Sigma‐Aldrich (S‐A); purity ≥ 99.5%), D‐(‐)‐fructose (S‐A; purity ≥ 99%), sucrose (S‐A; purity ≥ 99.5%), D‐(+)‐maltose monohydrated (S‐A; purity: ≥ 99%), starch (S‐A; ash: ≤ 0.4%), and cellulose (S‐A; ash: ~0.01%).

#### 2.1.2 Pretreatment and bromatological characterization

Samples were carefully dried in a vacuum oven (Fisher Scientific; Model 281A) heated at around 65°C. However, since the glucose, fructose, and sucrose samples exhibited a perceptible degradation process during such heating (e.g., browning and/or characteristic odor production), they were dried at vacuum conditions and room temperature. The dried samples were characterized, following the AOAC procedures (to avoid the humidification of samples, a careful handling was performed); the crude protein content was obtained by Kjeldahl method (Tests: 979.09; Official Methods of Analysis. AOAC International. Protein in grains, 2018a), considering a conversion factor of 5.83 g of protein/g of nitrogen (FAO Food and Nutrition, 2002), whereas the lipid content was measured by Soxhlet extraction (Test 920.39; Official Methods of Analysis. AOAC International. Fat (crude) or ether extract in animal feed, 2018b).

#### 2.1.3 Calorimetric characterization

The heat of combustion measurements were performed using an isoperibolic calorimeter, which included a combustion static bomb (Parr Model 1108; inner volume: 342 cm^3^); the used calorimeter has been described in more detail elsewhere (Orozco‐Guareño et al., 2019). The total calorific capacity of the calorimeter was determined by calibrating, using a certified standard of benzoic acid (certified heat of combustion: 6.318 ± 0.001 kcal/g) as the reference material and averaging 10 experimental measurements; benzoic acid samples used in these experiments were carefully handled to avoid any kind of contamination. Calorimetric measurements were performed in triplicate (using the same pattern sample), reporting as the representative value the mean value of the three experiments (${\text{HC}}_{\exp}$; expressed in kcal/g). The ${\text{HC}}_{\exp}$ value was corrected considering the relative amounts of lipids and proteins contained in the characterized samples, which were determined by means of the above mentioned test. The correction equation used is as follows:

(1) $$\text{HC}=\frac{{\text{HC}}_{\exp}-M_{l}\left(9.3\right)-M_{p}\left(5.8\right)}{M_{T}-M_{l}-M_{p}}$$

In Equation 1, HC is the corrected value of the heat of combustion (expressed in kcal/g), and *M_T, _M_l_* and *M_p_* are, respectively, the mass of the tested sample and the masses of the lipids and proteins contained in such sample (expressed in g). Equation 1 considers the heats of combustion reported by Atwater and Bryant (1900), that is, 9.3 kcal/g for the vegetable source lipids and 5.8 kcal/g for proteins contained in cereals.

To validate the respective “bibliographic intervals” with the corrected experimental data (i.e., the respective HC values), as in our previous work (Sánchez‐Peña et al., 2019), the boxplot (Iglewicz, 2011) and/or the Grubbs’ test (Grubbs, 1969; NIST/SEMATECH, 2012) were used to assign, or not, the considered data as “statistically outlier data.” Thus, the possible assignation of the experimental data (HC values) as “non‐outlier data” (Sánchez‐Peña et al., 2019) could be interpreted as a self‐validation of the two information sources, the bibliographic, and the experimental one. In that case, the “overall intervals” can be estimated following the same procedure used to determine the correspondent “bibliographic intervals” (Sánchez‐Peña et al., 2019), but considering as an additional datum of the each set of data the correspondent HC value. Since our experimental data were very carefully measured, following our parallel work (Sánchez‐Peña et al., 2019), they were considered type L‐III data. Henceforth, the respective “overall intervals” will be defined by means of their respective extreme values, that is, the $HC_{i}^{h}$ (the highest value of the “overall interval” for the carbohydrate i) and $HC_{i}^{l}$ (the lowest value of the “overall interval” for the carbohydrate i) values; from now on, for glucose, i=1; fructose, i=2; sucrose, i=3; maltose, i=4; starch, i=5; cellulose, i=6. Besides, the respective actualized representative values were estimated, like the correspondent representative values in our parallel paper (Sánchez‐Peña et al., 2019), but considering the correspondent experimental value (HC value) as an additional type L‐III datum.

To estimate the HC value of an anhydrous substance (${\text{HC}}_{\text{anh}}$), the following equation can be used (Sánchez‐Peña et al., 2019):

(2) $${\text{HC}}_{\text{anh}}{\;\text{= HC}}_{\text{hyd}}\left(\text{MH}\;/\;\text{MA}\right){\;\text{+ HH}}_{\text{substance}}$$

where ${\text{HC}}_{\text{anh}}$ and ${\text{HC}}_{\text{hyd}}$ are, respectively, the heats of combustion for the anhydrous and hydrated forms of the tested substance, whereas MH and MA are, respectively, the relative masses of the hydrated and anhydrous substance, finally, ${\text{HH}}_{\text{substance}}$ is the hydration heat of the tested substance. Due to crystallographic reasons, there are X + 18 g of hydrated substance by each X g of anhydrous substance and 18 g of water, being X the molar mass (expressed in g/mol) of the tested substance. Since the ${\text{HC}}_{\exp}$ value for the anhydrous maltose could not be experimentally determined, the equation 2 will be used to estimate the actualized representative value and the “overall interval” for such substance.

### 2.2 Estimation of the “global interval” for the heat of combustion and the global heat of combustion of the carbohydrate mass contained in a given food

#### 2.2.1 “Global interval” for the heat of combustion

##### Composition intervals

In the case of the carbohydrates contained in vegetal source foods, the information required to estimate the experimental interval for the respective compositions (${\bar{C}}_{i}$ and $se_{i}$ values; a pair for each carbohydrate contained in the considered food) was obtained from the USDA Food Composition Database (National Nutrient Database for Standard Reference, 2016), being such intervals estimable as follows (Montgomery, 2001):

(3) $$\left[{\bar{C}}_{i}-h_{i}\left(se_{i}\right)\sqrt{n},\;{\bar{C}}_{i}+h_{i}\left(se_{i}\right)\sqrt{n}\right]$$

where $h_{i}$ is the statistical factor assignable to each carbohydrate (1 ≤ i ≤ 6; see definitions above), which depend on the number of data measured (n value), the desired level of distribution coverage (here, a coverage of 90% was selected) and the defined confidence level (in this work, a 90% was chosen, assuming a distribution with two tail) (Montgomery, 2001), ${\bar{C}}_{i}$ are the respective mean values for the compositions of each carbohydrate contained in the different foods (expressed in mass percentage; 1 ≤ i ≤ 6; see definitions above), and $se_{i}$ are the respective standard errors of the experimental data used to calculate the corresponding ${\bar{C}}_{i}$ values (1 ≤ i ≤ 6; see definitions above). Alternatively, such intervals can be defined by means of their respective extreme values, that is, the $C_{i}^{h}$ ($={\bar{C}}_{i}+h_{i}\left(se_{i}\right)\sqrt{n}$) and $C_{i}^{l}$ ($={\bar{C}}_{i}-h_{i}\left(se_{i}\right)\sqrt{n}$) values; note that, when $se_{i}=0$, or this datum is not an available datum, $C_{i}^{h}=C_{i}^{l}={\bar{C}}_{i}$.

##### The “overall interval” for the heats of combustion

Equivalently to the indicated intervals in section “Composition intervals”, for each carbohydrate, there is an “overall interval” for the respective heats of combustion, which is defined by their respective extreme values, that is, the above defined ${\text{HC}}_{i}^{h}$ and ${\text{HC}}_{i}^{l}$ values (section 2.1.3.).

##### Estimating the “global interval” for the heat of combustion of the carbohydrates contained in a given food

When the six composition intervals (one for each carbohydrate type; section “Composition intervals”) and the six “overall intervals” for the heats of combustion (one for each carbohydrate type; section The “overall interval” for the heats of combustion) are available, it is possible to define an interval (the “global interval”) that very probably includes the heat of combustion produced by the carbohydrate mass contained in a given food.

To estimate such “global interval,” the following calculation procedure is proposed:

- Consider the extreme values of the six composition intervals (one interval for each carbohydrate type; *i *= 1, 2,…up to 6), that is, the six pairs of values $C_{i}^{h}$ and $C_{i}^{l}$; see section “Composition intervals”.
- Define 64 (=2^6^) different sets, each one containing six values (one for each carbohydrate type, that is, one for each value of i), utilizing all the combinations that result from selecting for each value of i, one of the two following values: $C_{i}^{h}$ or $C_{i}^{l}$. Please consider the following example: $\left\{C_{1}^{h},C_{2}^{h},C_{3}^{l},C_{4}^{h},C_{5}^{l},C_{6}^{h}\right\}$; in this set, the first term could be: $C_{1}^{h}$ or $C_{1}^{l}$, having been selected in this case $C_{1}^{h}$, the second one could be, $C_{2}^{h}$ or $C_{2}^{l}$, having been selected for this set $C_{2}^{h}$, and so on up to the last term, $C_{6}^{h}$, which was chosen between $C_{6}^{h}$ and $C_{6}^{l}$.
- Consider now the extreme values of the six “overall intervals” for the heats of combustion (one interval for each carbohydrate type, that is, for *i *= 1, 2,…up to 6), that is, the six pairs of values ${\text{HC}}_{i}^{h}$ and ${\text{HC}}_{i}^{l}$; see section The “overall interval” for the heats of combustion.
- Define 64 different sets (as in the penultimate point), each one containing six values (one for each carbohydrate type, that is, one for each value of *i*), utilizing all the combinations that result from selecting for each value of *i*, one of the following values: ${\text{HC}}_{i}^{h}$ or ${\text{HC}}_{i}^{l}$; the following is an example set: $\left\{{\text{HC}}_{1}^{l},{\text{HC}}_{2}^{h},{\text{HC}}_{3}^{l},{\text{HC}}_{4}^{h},{\text{HC}}_{5}^{l},{\text{HC}}_{6}^{l}\right\}$.
- Define now 4,096 (=64 × 64) different sets, each one containing six products (one for each value of i, that is, one for each carbohydrate contained in the studied food), utilizing all the combinations that result when each one of the 64 sets with composition values and each one of the 64 sets with heat of combustion values are considered. Example: $\left\{\left(C_{1}^{h}\right)\left({\text{HC}}_{1}^{l}\right),\left(C_{2}^{h}\right)\left({\text{HC}}_{2}^{h}\right),\left(C_{3}^{l}\right)\left({\text{HC}}_{3}^{l}\right),\left(C_{4}^{h}\right)\left({\text{HC}}_{4}^{h}\right),\left(C_{5}^{l}\right)\left({\text{HC}}_{5}^{l}\right),\left(C_{6}^{h}\right)\left({\text{HC}}_{6}^{l}\right)\right\}$; note that, in this set, the previously proposed example sets for composition and for heat of combustion were used.
- Estimate the 4,096 possible extreme global values for the heat of combustion of the total carbohydrate mass contained in a given food, by summing the six products indicated in each one of the 4,096 sets of six products defined in the previous point and dividing each resulting number over 100 (or, equivalently, multiplying by 0.01). Example: ${\text{HC}}_{c}^{f}{|}_{x}=0.01\left\{\left(C_{1}^{h}\right)\left({\text{HC}}_{1}^{l}\right)+\left(C_{2}^{h}\right)\left({\text{HC}}_{2}^{h}\right)+\left(C_{3}^{l}\right)\left({\text{HC}}_{3}^{l}\right)+\left(C_{4}^{h}\right)\left({\text{HC}}_{4}^{h}\right)+\left(C_{5}^{l}\right)\left({\text{HC}}_{5}^{l}\right)+\left(C_{6}^{h}\right)\left({\text{HC}}_{6}^{l}\right)\right\}$ being ${\text{HC}}_{c}^{f}{|}_{x}$, the x‐esim possible value for the heat of combustion of the carbohydrate mass contained in the food *f*.
- Define the “global interval” for the heat of combustion, considering that such interval must include exactly the 4,096 individual values calculated in  the previous point. Therefore, the “global interval” for the heat of combustion ranges from the minimum value of the list of the 4,096 values for the ${\text{HC}}_{c}^{f}{|}_{x}$ parameter to the maximum one of such list; thus, it could be expected that the real value of the global heat of combustion is within such interval.

#### 2.2.2 Global heat of combustion

When a simplified vision is used, the global heat of combustion of the carbohydrate mass contained in a given food (${\text{HC}}_{\mathrm{cinf}}$; expressed in kcal/g of carbohydrate in such food) could be estimated by means of the following equation (Sánchez‐Peña et al., 2019).

(4) $${\text{HC}}_{\mathrm{cinf}}=0.01\left[\left({\bar{C}}_{1}\right)\left({\text{HC}}_{1}\right)+\left({\bar{C}}_{2}\right)\left({\text{HC}}_{2}\right)+\left({\bar{C}}_{3}\right)\left({\text{HC}}_{3}\right)+\left({\bar{C}}_{4}\right)\left({\text{HC}}_{4}\right)+\left({\bar{C}}_{5}\right)\left({\text{HC}}_{5}\right)+\left({\bar{C}}_{6}\right)\left({\text{HC}}_{6}\right)\right]$$

where ${\bar{C}}_{i}$ is the mean percentage composition reported for the respective carbohydrate *i* in a given food (e.g., those values reported for the USDA Food Composition Database (National Nutrient Database for Standard Reference, 2016)), and ${\text{HC}}_{i}$ is the actualized representative value for the heat of combustion of the corresponding carbohydrate (estimated according to section 2.1.3.).

However, when the information required to estimate the “global interval” for the heat of combustion of the carbohydrates contained in a given food is available (section “Estimating the “global interval” for the heat of combustion of the carbohydrates contained in a given food”), an alternative option to estimate the global heat of combustion representative of the carbohydrate mass contained in such food (${\text{HC}}_{c}^{f}$), is to average the 4,096 ${\text{HC}}_{c}^{f}{|}_{x}$ values (section “Estimating the “global interval” for the heat of combustion of the carbohydrates contained in a given food”). Note that, although the ${\text{HC}}_{c}^{f}$ value could a priori be considered equivalent to the ${\text{HC}}_{\mathrm{cinf}}$ value, the statistical superiority of the first one is evident; numerical comparisons on this topic will be presented below.

For this study, a computational program was specifically designed to perform the calculation procedures described in sections “Estimating the “global interval” for the heat of combustion of the carbohydrates contained in a given food” and 2.2.2.

## 3 RESULTS

The *HC* values of glucose, fructose, sucrose, monohydrated maltose, starch, and cellulose, obtained by correcting (Equation 1) the respective experimental values (${\text{HC}}_{\exp}$) are the following (expressed in kcal/g): (a) glucose: 3.759, (b) fructose: 3.790, (c) sucrose: 3.987, (d) monohydrated maltose: 3.764, (e) starch: 4.142, and (f) cellulose: 4.117. By applying the suitable statistical test (Grubbs, 1969; Iglewicz, 2011; NIST/SEMATECH, 2012), it was demonstrated that the HC values are not outlier, when compared with the respective available bibliographic information (Sánchez‐Peña et al., 2019).

Thus, using the procedure previously indicated (section 2.1.3.), the following “overall intervals” were calculated (expressed in kcal/g): (a) glucose: 3.69–3.78, (b) fructose: 3.70–3.81, (c) sucrose: 3.91–4.00, (d) monohydrated maltose: 3.71–3.81, (e) starch: 4.07–4.25, and (f) cellulose: 4.05–4.25. Due to it is required estimating the “overall interval” for anhydrous maltose, this interval was estimated considering the Equation 2, the respective extreme values of the “overall interval” estimated for the monohydrate maltose (3.71 and, 3.81 kcal/g), and the ${\text{HH}}_{\text{maltose}}$ value used in a parallel report (${\text{HH}}_{\text{maltose}}≅0.03\text{kcal/g}$; Sánchez‐Peña et al., 2019), resulting in the following “overall interval” for the anhydrous maltose: 3.93–4.04 kcal/g.

Regarding the respective actualized representative values (section 2.1.3.), the obtained results are the following (expressed in kcal/g): (a) glucose: 3.74, (b) fructose: 3.76, (c) sucrose: 3.95, (d) monohydrated maltose: 3.76, (e) starch: 4.16, and (f) cellulose: 4.15. The actualized representative value for anhydrous maltose was estimated from the equivalent datum for the monohydrated maltose (3.76 kcal/g), the ${\text{HH}}_{\text{maltose}}$ value previously mentioned (Sánchez‐Peña et al., 2019), and the use of the Equation 2, obtaining the following value: 3.99 kcal/g of anhydrous maltose.

In Tables 1, 2, 3, the global heats of combustion (${\text{HC}}_{c}^{f}$), the global intervals of the heat of combustion (global intervals), and the respective ${\text{HC}}_{\mathrm{cinf}}$ values corresponding to the considered foods are shown; Table 1 presents the results corresponding to the 31 considered fruits, Table 2 shows the values calculated for the 24 vegetables studied, and Table 3 includes the values estimated for 13 types of cereal samples.

**Table 1:** Global calorimetric information about carbohydrates contained in fruits

No.	Food description	Global values		Global intervals
		${\text{HC}}_{c\;inf}$	${\text{HC}}_{c}^{f}$	
1	Apples raw fuji, with skin	3.85	3.84	3.73–3.97
2	Apples raw gala, with skin	3.87	3.87	3.78–3.96
3	Apples raw golden delicious	3.86	3.86	3.78–3.94
4	Apples raw granny smith with skin	3.87	3.87	3.79–3.96
5	Apples raw red delicious with skin	3.85	3.85	3.75–3.96
6	Blueberries, raw	3.83	3.83	3.73–3.98
7	Cherries, sour, red, raw	3.82	3.82	3.75–3.92
8	Clementines raw	3.92	3.91	3.79–4.01
9	Currants red and white raw	3.91	3.92	3.79–4.09
10	Dates, deglet noor	3.86	3.86	3.75–3.96
11	Dates medjool^a^	3.78	3.78	3.73–3.84
12	Figs dried uncooked	3.84	3.84	3.77–3.92
13	Grapefruit raw pink and red, all areas	3.91	3.90	3.76–4.08
14	Grapes, muscadine raw^a^	3.89	3.89	3.82–3.95
15	Grapes red or green, raw (European type)	3.77	3.77	3.70–3.85
16	Jackfruit raw^a^	3.80	3.80	3.75–3.86
17	Kiwi fruit green raw	3.85	3.85	3.74–3.97
18	Lemon juice, raw	3.85	3.84	3.69–3.98
19	Lime juice, raw	3.87	3.88	3.76–4.00
20	Melons honeydew raw	3.86	3.85	3.69–4.01
21	Nectarines, raw	3.93	3.93	3.80–4.06
22	Peaches yellow, raw	3.91	3.92	3.81–4.03
23	Pears raw	3.86	3.86	3.76–3.97
24	Pears raw bartlett	3.86	3.86	3.75–3.97
25	Pears raw bosc	3.88	3.88	3.75–4.03
26	Pears raw green Anjou	3.85	3.86	3.75–3.99
27	Pears raw red Anjou	3.85	3.85	3.74–3.98
28	Pineapple raw, all varieties	3.91	3.90	3.79–4.00
29	Pineapple raw extra sweet variety	3.91	3.91	3.78–4.02
30	Plums raw	3.83	3.83	3.72–3.94
31	Rowal raw^a^	4.00	4.00	3.94–4.06

^a^ For these foods, the global interval was estimated considering, for each carbohydrate, the only available composition value or, otherwise, such interval was calculated considering the mean of the two composition values available for each carbohydrate; the last action was required, since neither the individual values nor the corresponding *se* value were reported.

**Table 2:** Global calorimetric information about carbohydrates contained in vegetables

No.	Food description	Global values		Global intervals
		${\text{HC}}_{c\;in\;f}$	${\text{HC}}_{c}^{f}$	
1	Alfalfa seed, sprouted, raw	4.07	4.08	3.93–4.25
2	Brussels sprouts, raw^a^	4.02	4.02	3.94–4.10
3	Cabbage, raw	3.93	3.93	3.84–4.02
4	Cabbage, red, raw	3.91	3.91	3.79–4.05
5	Carrots, baby, raw	3.97	3.97	3.83–4.13
6	Carrots, raw	4.01	4.02	3.90–4.19
7	Cucumber, with peel, raw	3.93	3.94	3.82–4.11
8	Lettuce, green leaf, raw	4.00	4.00	3.87–4.16
9	Lettuce, iceberg (includes crisphead types), raw	3.90	3.91	3.77–4.08
10	Mushrooms, chanterelle, raw^a^	4.05	4.05	3.97–4.14
11	Mushrooms, maitake, raw^a^	3.99	3.99	3.91–4.07
12	Mushrooms, portabella, raw	3.88	3.87	3.74–4.06
13	Okra, raw	4.05	4.06	3.95–4.16
14	Onions, raw	3.89	3.90	3.79–4.01
15	Onions, sweet, raw	3.83	3.83	3.74–3.94
16	Onions, yellow, sauteed	3.89	3.89	3.77–4.02
17	Peppers, jalapeno, raw	3.91	3.90	3.77–4.03
18	Peppers, sweet, green, raw	3.92	3.92	3.82–4.03
19	Peppers, sweet, red, raw	3.88	3.89	3.79–4.01
20	Radishes, raw	3.94	3.95	3.81–4.12
21	Rutabagas, raw^a^	3.91	3.91	3.85–3.98
22	Spinach, raw	4.07	4.07	3.93–4.25
23	Squash, summer, zucchini, includes skin, raw	3.87	3.87	3.74–4.04
24	Squash, winter, butternut, raw^a^	3.95	3.95	3.88–4.02

^a^ For these foods, the global interval was estimated considering, for each carbohydrate, the only available composition value or, otherwise, such interval was calculated considering the mean of the two composition values available for each carbohydrate; the last action was required, since neither the individual values nor the corresponding *se* value were reported.

**Table 3:** Global calorimetric information about carbohydrates contained in cereals

No.	Food description	Global values		Global intervals
		${\text{HC}}_{c\;in\;f}$	${\text{HC}}_{c}^{f}$	
1	Amaranth grain, uncooked	4.15	4.15	4.05–4.25
2	Cornmeal, degermed, enriched, white	4.14	4.14	4.04–4.25
3	Pasta, dry, unenriched	4.15	4.15	4.06–4.24
4	Pasta, gluten‐free, corn and rice flour, cooked	4.16	4.16	4.07–4.25
5	Pasta, whole‐wheat, cooked	4.15	4.15	4.06–4.24
6	Potatoes, flesh, and skin, raw	4.14	4.13	3.99–4.25
7	Potatoes, red, flesh and skin, raw	4.13	4.14	4.03–4.25
8	Potatoes, white, flesh and skin, raw	4.13	4.13	4.04–4.23
9	Rice, brown, long‐grain, raw	4.16	4.16	4.07–4.25
10	Rye flour, light	4.12	4.11	3.99–4.25
11	Rye grain	4.12	4.12	4.01–4.25
12	Sorghum flour, refined, unenriched^a^	4.09	4.09	4.00–4.18
13	Spelt, uncooked	4.13	4.13	4.02–4.25

^a^ for this food, the global interval was estimated considering, for each carbohydrate, the only available composition value.

**Figure 1:**
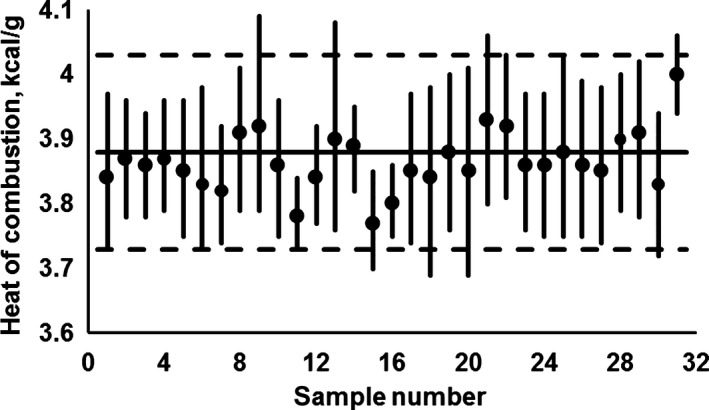
Global intervals for the heat of combustion of the carbohydrates contained in different types of fruits (**‐**), and their correspondent ${\text{HC}}_{c}^{f}$ values (●); the sample number correspond to the “No.” indicated in Table 1. The proposed interval to represent the set of global intervals is limited by dotted lines and its central line is represented by the continuous horizontal line (see additional information in text)

**Figure 2:**
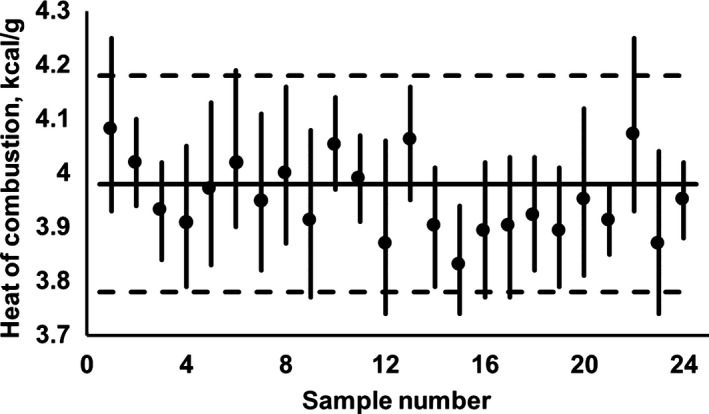
Global intervals for the heat of combustion of the carbohydrates contained in different types of vegetables (**‐**), and their correspondent ${\text{HC}}_{c}^{f}$ values (●); the sample number correspond to the “No.” indicated in Table 2. The proposed interval to represent the set of global intervals is limited by dotted lines and its central line is represented by the continuous horizontal line (see additional information in text)

**Figure 3:**
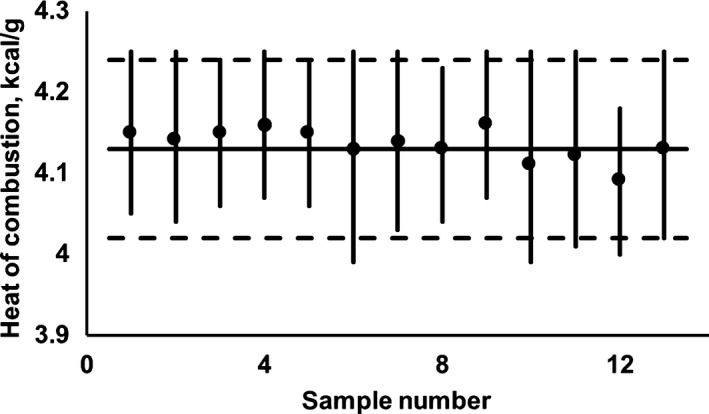
Global intervals for the heat of combustion of the carbohydrates contained in different types of cereals (**‐**), and their correspondent ${\text{HC}}_{c}^{f}$ values (●); the sample number correspond to the “No.” indicated in Table 3. The proposed interval to represent the set of global intervals is limited by dotted lines and its central line is represented by the continuous horizontal line (see additional information in text)

In addition, to facilitate the analysis of the global intervals of the samples corresponding to the same type of food (fruits, vegetables, or cereals), Figures 1, 2, 3 are presented; the information about fruits was included in Figure 1, the data referring to vegetables were presented in Figure 2 and, finally, the values regarding cereals were showed in Figure 3.

## 4 DISCUSSION

Since the respective HC values could not be statistically classified as “outlier data” (Grubbs,^1969^; Iglewicz, 2011; NIST/SEMATECH, 2012), the existence of a high level of agreement among the correspondent bibliographic information (Sánchez‐Peña et al., 2019) and the respective HC values can be recognized, which reinforce the validity of both sources.

Moreover, as it could be expected from this agreement, when the “overall intervals” are compared with the corresponding “bibliographic intervals” (Sánchez‐Peña et al., 2019), only marginal differences in the respective extreme values could be evidenced; in the most of cases, approximately 0.01 kcal/g. Similarly, when our results are compared with the respective representative values bibliographically obtained (Sánchez‐Peña et al., 2019), it can be affirmed that, in general terms, the inclusion of the correspondent experimental datum in each set of data, produced negligible modifications in the respective representative values (approximately 0.01 kcal/g).

When comparing, food by food, the respective ${\text{HC}}_{c}^{f}$ and ${\text{HC}}_{\mathrm{cinf}}$ values presented in Tables 1, 2, 3, it can be noticed that, in all the foods considered, the respective differences between them can be considered negligible (for each food, $\left|{\text{HC}}_{c}^{f}-{\text{HC}}_{\mathrm{cinf}}\right|\leq 0.01\text{kcal/g}$).

Thus, although the ${\text{HC}}_{c}^{f}$ values will henceforth be considered as the more accurate representative values for the correspondent foods, considering the high level of agreement between the respective ${\text{HC}}_{c}^{f}$ and ${\text{HC}}_{\mathrm{cinf}}$ values, when the estimation of the ${\text{HC}}_{c}^{f}$ value implies a considerable operative difficulty, such value can acceptably be substituted by the ${\text{HC}}_{\mathrm{cinf}}$ value.

Regarding the global intervals, the first aspect that should be mentioned is related with the corresponding “interval lengths” (e.g., the maximum value minus the minimum one of the respective global interval). About this topic, although the USDA Food Composition Database (National Nutrient Database for Standard Reference, 2016) lists the contents of the different carbohydrates present in numerous foods, for some of them, the reported se values were comparatively high, which produced “interval lengths” that were herein considered too long. In this work, the “interval lengths”> 0.32 kcal/g were considered too long, therefore, the foods that fulfill this condition were not considered in this report. Taking into account only the 68 foods considered in Tables 1, 2, 3, only in five of them, the “interval lengths” were in the proposed limit value (0.32 kcal/g or, equivalently, ±4%).

Thus, looking for a estimation of the inaccuracy level expectable to use the ${\text{HC}}_{\mathrm{cinf}}$ parameter as a representative value for a given food (its calculation facility makes it a “very attractive” parameter) instead of the correspondent global interval, in Tables 1, 2, 3 it can be noticed that, in the vast majority of considered foods (87%), the correspondent global intervals are within the generic interval ${\text{HC}}_{\mathrm{cinf}}\pm 4\%$; only in nine foods, one of the extremes of the individual interval exceeded to the generic interval, falling out of it around 0.01 kcal/g. Based on the previous results, it could be affirmed that the expectable error to consider as representative of the calorimetric behavior of a given food to its ${\text{HC}}_{\mathrm{cinf}}$ value is ≤4%; by its evident calculation facility, at least in some cases, this could be an attractive option to approximate the calorimetric behavior in a given food.

To facilitate the analysis of the global intervals of the samples corresponding to the same type of food (fruits, vegetables, or cereals), Figures 1, 2, 3 are presented. Thus, Figure 1 includes the available information for fruits, being there possible to appreciate that the 31 global intervals can be suitably represented by the following globalizing mathematical expression: 3.88 kcal/g ± 4%; such expression is graphically represented by the horizontal lines in Figure 1. The previous expression was *ex‐profeso* calculated to “exclude,” in both extremes (superior and inferior), less than 2.5% of the “global interval length” (calculated as the arithmetic sum of the 31 “interval lengths”).

Per the above, and considering the available data for fruits (Figure 1), it can here be generalized that: ${\text{HC}}_{c}^{\text{fruits}}=3.88\text{kcal/g}\pm 4\%$. When comparing this value with the equivalent one proposed by Atwater for vegetable source foods (${\text{HC}}_{c}=4.2\text{kcal/g}$), it is evident that an overestimation that ranges between 4% and 11% is involved when Atwater's value is used.

In Figure 2, the available information relative to the considered vegetables is presented. Then, after an equivalent analysis to the previously described, it was found that the mathematical expression suitable for vegetables is as follows: ${\text{HC}}_{c}^{\text{vegetables}}=3.98\text{kcal/g}\pm 5\%$; in this type of foods, the use of Atwater's value (4.2 kcal/g) involves an overestimation on the heat of combustion value that can become as high as 10%.

Finally, when the available results for cereals are analyzed in Figure 3, it can be proposed that the representative expression for this type of foods is as follows: ${\text{HC}}_{c}^{\text{cereals}}=4.13\text{kcal/g}\pm 3\%$. As in previous food groups (fruits and vegetables), the use of Atwater's value involves an overestimation of the correspondent heat of combustion value, which, in this specific food group can become as high as 5%.

## 5 CONCLUSIONS

The mutual consistency between the experimental results determined herein and the bibliographic data collected in our parallel work (Sánchez‐Peña et al., 2019) allow us to consider them as experimentally validated, allowing its suitable use in later calculations. In addition, the obtained generalized intervals demonstrate that, in the case of vegetable source foods, the use of Atwater's value (4.2 kcal/g of carbohydrate) involves a clear overestimation of the heat of combustion of the carbohydrates contained in such type of foods, which can become as high as 11%. Finally, it is clear that the proposed set of ${\text{HC}}_{c}$ values represents a more specific, accurate, and precise set of values than the global value proposed by Atwater for the heat of combustion of the carbohydrates contained in vegetable source foods, but, besides (and maybe mainly), represents a set of values much more supported/argued from the experimental and statistical point of views.

## CONFLICT OF INTEREST

Authors declare that they do not have any conflict of interest.

## ETHICAL REVIEW

This study does not involve any human or animal testing.

## INFORMED CONSENT

Does not apply.
